# High Prevalence of *Clostridium difficile* Colonization among Nursing Home Residents in Hesse, Germany

**DOI:** 10.1371/journal.pone.0030183

**Published:** 2012-01-11

**Authors:** Mardjan Arvand, Vera Moser, Christine Schwehn, Gudrun Bettge-Weller, Marjolein P. Hensgens, Ed J. Kuijper

**Affiliations:** 1 Hesse State Health Office, Centre for Health Protection, Dillenburg, Germany; 2 Department of Medical Microbiology, Leiden University Medical Center, Leiden, The Netherlands; Charité, Campus Benjamin Franklin, Germany

## Abstract

*Clostridium difficile* is the most common cause of antibiotic-associated diarrhoea in hospitals and other healthcare facilities. The elderly are particularly susceptible and at increased risk for adverse outcome as a result of *C. difficile* infection. The aim of this study was to determine the prevalence of *C. difficile* colonization among residents of nursing homes in Hesse and to compare it with the prevalence in the general population living outside long-term care facilities (LTCF). We assessed possible risk factors for *C. difficile* colonization and determined the genotype of circulating strains. *C. difficile* was isolated from 11/240 (4.6%) nursing home residents and 2/249 (0.8%) individuals living outside LTCF (p = 0.02). Ten of 11 (90.9%) isolates from nursing homes and one of two isolates from the population outside LTCF were toxigenic. The prevalence of *C. difficile* colonization varied from 0% to 10% between different nursing homes. Facilities with known actual or recent CDI cases were more likely to have colonized residents than facilities without known CDI cases. *C. difficile* PCR-ribotypes 014 and 001 were the most prevalent genotypes and accounted for 30% and 20% of toxigenic isolates in nursing homes, respectively. Interestingly, no individuals carried the epidemic strain PCR-ribotype 027. Our results suggest that residents of nursing homes in Germany are at high risk for colonization by virulent *C. difficile* strains. The high prevalence of *C. difficile* colonization in nursing homes underscores the importance of good adherence to standard infection control precautions even in the absence of a diagnosed infection. They also emphasize the need for specific programs to increase the awareness of healthcare professionals in LTCF for CDI.

## Introduction

*Clostridium difficile* infection (CDI) caused by an anaerobic, gram-positive, spore-forming bacillus is the most common cause of healthcare-associated infectious diarrhoea in healthcare facilities. There is a strong association between antimicrobial therapy and CDI, as *C. difficile* can only colonize the gut if the normal intestinal flora is disturbed or absent [1]. The incidence and severity of CDI has markedly increased over the last 10–15 years. This has been attributed to multiple factors including changing demographic situation, increased use of broad-spectrum antibiotics and emergence of hypervirulent *C. difficile* strains [2].

The elderly are particularly susceptible and at increased risk for adverse outcomes as a result of CDI [3], [4]. The increased risk of acquiring *C. difficile* infection in the elderly may be due to age-related changes in faecal flora, immune senescence, or the presence of other underlying diseases [4]. Recently, several outbreaks of CDI have been reported from nursing homes in different European countries and the USA [5], [6], [7], [8]. Little is known about the incidence, prevalence, and molecular epidemiology of CDI in nursing homes in the absence of an epidemic situation [9], [10]. To our knowledge, this is the first study on prevalence of *C. difficile* among nursing home residents in Germany.

In this survey, we studied the prevalence of *C. difficile* colonization among residents of different nursing homes in Hesse, a state with approximately six Million inhabitants located in Southwest Germany. For comparison, we determined the rate of *C. difficile* colonization in the general population living outside nursing homes in the same geographic region. We evaluated which factors were associated with *C. difficile* colonization. The *C. difficile* isolates were tested for toxin production and further characterized by PCR-ribotyping to determine their genetic relationship and to evaluate the distribution of epidemic genotypes.

## Results

**Figure 1:**
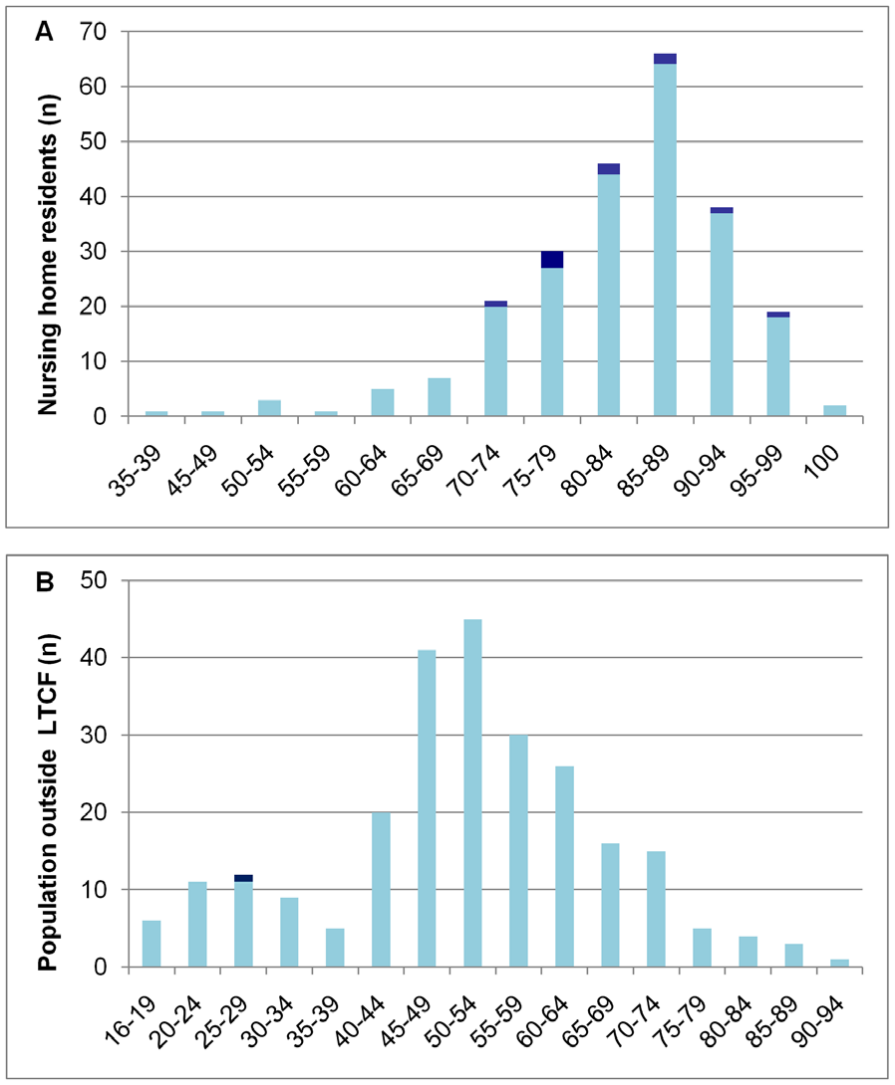
Age structure of participants from nursing home (A) and general population (B). Participants colonized by toxigenic *C. difficile* are shown in dark blue.

Using a cross-sectional design, we studied the prevalence of intestinal colonization by *C. difficile* among 240 nursing home residents and 249 volunteers living outside LTCF in Hesse. A summary of demographic and anamnestic information of the participants is presented in Table 1. The groups differed in terms of age, with a mean age of 83 years in nursing home residents versus 51 years in the population outside LTCF (Figure 1). The majority of participants were female. The history of hospital admission and antibiotic therapy during previous three months, and prevalence of diarrhoea at study time were similar between nursing home residents and the population outside LTCF (Table 1).

**Table 1:** Summary of Demographic and anamnestic information about the participants from nursing homes and the general population outside LTCF.

Condition	Nursing home residents	General population	p
Age, range, yr	38–100	16–90	<0.01
Age, mean, yr	83	51	<0.01
Age, median, yr	85	52	<0.01
Male, n/total* (%)	52/239 (21.8)	97/248 (39.1)	<0.01
Hospital Admission during previous three months, n/total* (%)	35/240 (14.6)	30/226 (13.3)	0.68
Antimicrobial therapy during previous three months, n/total* (%)	40/240 (16.7)	32/226(14.2)	0.45
Diarrhoea at study time, n/total* (%)	7/240 (2.9)	5/226 (2.2)	0.63

*Total number of participants with available information.

*C. difficile* was isolated from 11 (4.6%) of 240 nursing home residents and two (0.8%) of 249 participants living outside LTCF (p = 0.02). Ten (90.9%) of 11 isolates obtained from the nursing home residents and one of two isolates from the population outside LTCF were toxigenic, as determined by ELISA and PCR. Thus, ten (4.2%) of 240 nursing home residents and one (0.4%) of 249 participants from outside LTCF were colonized by toxigenic *C. difficile* isolates (p = 0.01).

The prevalence of *C. difficile* colonization varied from 0% to 10% between different nursing homes (Table 2). The colonization rate was 10% in two facilities and 9.1% in another nursing home. Molecular characterization of the isolates revealed that the PCR-ribotypes (RT) 014 and 001 were the most prevalent genotypes in nursing homes, accounting for 30% and 20% of toxigenic isolates, respectively. Other ribotypes including 015, 045, 046, RKI-57, 216 were detected only once each. We did not find any case of colonization by the presumably hypervirulent strains RT 027 or 078 in this study. When two or three residents in one facility were colonized by *C. difficile*, the associated isolates were always assigned to different ribotypes (Table 2).

**Table 2:** Prevalence of *C. difficile* colonization and characteristics of the isolates obtained from 240 nursing home residents in Hesse, Germany.

Nursing home	Specimens examined	*C. difficile* positive, n (%)	Toxigenic culture positive, n (%)	PCR-ribotype	CDI case in facility at study time	CDI case in facility during previous 6 months	Specific infection control guidance available for CDI
**A**	39	1 (2.6)	1 (2.6)	046	yes	yes	no
**B**	16	1 (6.3)	1 (6.3)	001	no	no	yes
**C**	17	1 (5.9)	0	031	no	no	yes
**D**	30	3 (10)	3 (10)	014, 045, RKI-57	no	yes	yes
**E**	10	1 (10)	1 (10)	001	no	yes	no
**F**	24	1 (4.2)	1 (4.2)	015	no	no	yes
**G**	12	0	0		no	no	yes
**H**	28	0	0		no	no	yes
**I**	22	2 (9.1)	2 (9.1)	014, 216	no	yes	no
**J**	23	1 (4.3)	1 (4.3)	014	no	no	yes
**K**	19	0	0		no	no	yes
**Total**	**240**	**11 (4.6)**	**10 (4.2)**		**1 (9)**	**4 (36)**	**8 (73)**

The toxigenic *C. difficile* isolate from the population outside LTCF was assigned to RT 070. It was recovered from a 28-years-old woman who had received three cycles of antimicrobial therapy for recurrent urinary tract infection. She had no history of recent hospital admission and had no diarrhoea at the time of sample collection.

Factors associated with *C. difficile* colonization of nursing home residents were evaluated by comparing colonized residents with those who were not colonized (Table 3). There was no apparent association between colonization and age, contact within previous four weeks with a person with diarrhoea, dementia, and incontinence (urine or feces). Previous CDI, previous antibiotic therapy and previous hospital admission were significantly associated with colonization by toxigenic *C. difficile* in nursing home residents (p≤0.01, Table 3).

**Table 3:** Characteristics of the nursing home residents in correlation with colonization by toxigenic *C. difficile*.

Characteristics	Colonized (n = 10)	Not colonized (n = 230)	Prevalence ratio (95% CI)	P
Age (mean)	83.0	82.5	–	0.86
Previous CDI	1	1	13.2 (2.87–62.5)	<0.01
Residence in a nursing home with CDI among residents during previous six months	7	94	3.22 (0.85–12.0)	0.08
Contact to patients with diarrhoea	0	10	–	–
Infection/colonization by MRSA or other multiresistant organisms	0	7	–	–
Antibiotic therapy in previous three months	5	35	5.00 (1.52–16.4)	0.01
Hospital admission during previous three months	5	30	5.85 (1.79–19.2)	<0.01
Chronic wound	1	8	2.67 (0.37–19.2)	0.33
Percutaneous-oesophageal-gastrotomy feeding tube	2	18	2.75 (0.63–12.0)	0.18
Dementia	4	121	0.61 (0.18–2.12)	0.45
Incontinence, urine	7	181	0.65 (0.17–2.41)	0.53
Incontinence, feces	5	120	0.92 (0.27–3.10)	0.90

The data were evaluated by comparing colonized to not colonized residents and calculating the point prevalence ratio.

Together, colonization by toxigenic *C. difficile* was observed among residents of seven of eleven nursing homes (Table 2). According to the information obtained by the questionnaire about structure and history of CDI in the nursing home, there was only one case of CDI known in one facility (nursing home A) at the time of sample collection. CDI cases at the sampling time or during the previous six months were reported from four facilities (nursing homes A, D, E, I; seven residents). All these facilities were tested positive for colonized residents (Table 2). Seven facilities had reported no actual or recent cases of CDI. Among these, three were positive for residents colonized by toxigenic isolates, and four were negative. According to the information obtained from the facilities' care management, specific infection control and management guidance for CDI were available in eight nursing homes (Table 2).

## Discussion

The aim of this study was to determine the prevalence and molecular epidemiology of *C. difficile* colonization among nursing home residents in Hesse, and to compare it with the general population living outside LTCF in the same geographic region. It is noteworthy that latter group did not contain elderly people only, but was representative for the adult population of Hesse. The proportion of elderly individuals (≥65 years) was 17.7% in our study. In comparison, according to the population pyramid age structure, 19.7% of the population in Hesse was ≥65 years in 2008 (Figure S1). Taking into account that a considerable proportion of elderly people lives in nursing homes in Germany (and is thus encountered in the nursing home group), we believe that the composition of the control group is realistic in our study. We found a similar rate of colonization by avirulent *C. difficile* isolates in both groups (0.4%). In contrast, colonization by toxigenic *C. difficile* isolates was ten-times higher in nursing home residents than in the population outside LTCF (4.2% versus 0.4%, p = 0.01). These results suggest that (i) nursing home residents are more likely to be colonized by *C. difficile* than the general population outside LTCF, and (ii) nursing home residents are preferentially exposed to toxigenic *C. difficile* strains as compared with population outside LTCF.

Increasing age, recent exposure to antimicrobial agents, and recent hospital admission have previously been described as risk factors for acquisition of *C. difficile* and development of CDI [1], [4], [9], [11], [12], [13], [14]. In our study, the groups differed in terms of age, which is not surprising, because the average age is currently 45 years in the general population in Germany (Figure S1), whereas nursing home residents are mostly elderly individuals. They did not differ in terms of history of previous antibiotic therapy or hospital admission, which is surprising at first glance. This may be due to the composition of the control group which also included individuals who attended a general practitioner's practice or a pharmacy. However, the percentage of individuals enrolled by the latter route was less than 10%. Another possible explanation is that people with potential risk factors such as antibiotic pre-treatment and prior hospitalisation were more easily motivated to participate in this study. Nonetheless, this does not seem to have a noticeable impact on the outcome, since the prevalence of *C. difficile* in the population outside LTCF was very low. In summary, our results indicate that the combination of high age and living in a nursing home is a significant risk factor for colonization by toxigenic *C. difficile*. However, since our control group included the entire adult population and not only elderly people living outside nursing homes, we can not asses the influence of the factors high age and living in a nursing home alone.

Our data are in accordance with studies from the United Kingdom and the USA, which revealed a prevalence of 0–20% for *C. difficile* colonization in LTCF in the absence of a recognized outbreak [4], [9], [15]. Our finding of 0.8% colonization rate among individuals outside a healthcare facility is lower as recently reported in a community-dwelling elderly population in the United Kingdom [16]. In that study, 6 (4%) of 149 samples were positive, but five of six positive samples were detected by enrichment culture only. We did not apply an enrichment culture and our results may therefore underestimate both carriership in residents of nursing homes and individuals outside LTCF.

Various factors may influence the prevalence of *C. difficile* in nursing homes, e.g. differences in the debility of residents, antibiotic consumption, hospital admission rate, infection control practise, strain virulence, and in alertness and preparedness of the facilities for CDI. We found that facilities with actual or recent cases of CDI were more likely to have colonized residents than those without known CDI cases. Previous studies have shown that shedding of *C. difficile* may persist for several weeks after resolution of diarrhoea [17]. It is estimated that 15–20% of CDI patients may experience a recurrence, resulting in prolonged carriership and shedding [18]. In addition, symptomatic patients with CDI who remain in the nursing home may represent a source of infection. In this context, it is important to notice that only 73% of the nursing homes in our study had specific infection control and management guidance for CDI, suggesting that additional efforts are required to further improve the infection control management in nursing homes in Germany.

Nine different PCR-ribotypes were obtained from the nursing home residents in this study. The most common ribotypes were 014 and 001, which belong to the most prevalent genotypes among hospitalized patients with CDI in Germany and in Europe [19], [20]. We did not detect isolates from the epidemic RT 027 and 078 in this study, although these strains have been repeatedly isolated from hospitalized patients with CDI in Hesse and in Germany [19], [21], [22], [23]. It can be hypothesized that PCR-ribotypes 027 and 078 might be rather associated with infection than colonization in Germany. However, since the number of isolates collected in this study was limited, our findings are probably not representative for the distribution of *C. difficile* genotypes in nursing homes in Germany. Further investigation with a larger panel of isolates is required to evaluate this hypothesis.

The present study is the first survey on prevalence of *C. difficile* colonization among nursing home residents in Germany. Strengths of the study are the inclusion of residents of eleven nursing homes and a control group representing the general population outside LTCF. Second, all stool samples were cultured and *C. difficile* isolates were characterized by toxin assays and molecular typing. Our study has also a few limitations. First, although approximately 500 individuals were enrolled, the number of collected isolates was rather limited. Second, only 44% of the nursing homes that were asked for participation agreed and were enrolled. Third, the percentage of residents that participated in this study varied between 15% and 48% in different nursing homes (median 31.5%), because not all residents or their legal guardians agreed with participation, some residents were not cooperative in terms of collection of stool samples, and some healthcare professionals were not interested. Therefore, it is likely that our prevalence data are not representative for all nursing home residents in Germany, but present a broad outline of the circulation of *C. difficile* in nursing homes. Fourth, we do not have exact data about the incidence of CDI in the nursing homes. It is noteworthy that medical care for nursing home residents is not centrally organised by the facility's management in Germany. Each resident has his or her own general practitioner, who is in charge of diagnostic and therapy. This may lead to differences within a nursing home and also between different facilities with regard to the frequency of conduction and choice of diagnostic tests for *C. difficile*.

In summary, our results demonstrate that nursing home residents in Hesse are at high risk for colonization by toxigenic *C. difficile*. The high prevalence of *C. difficile* colonization in nursing homes underscores the importance of good adherence to standard infection control precautions even in the absence of a diagnosed infection. They also emphasize the need for specific programs to increase the awareness of healthcare professionals in LTCF for CDI.

## Materials and Methods

### Study population

Twenty-five nursing homes located in 20 districts in Hesse were invited by telephone call, e-mail, and letter to participate in this study. Participation conditions were: i) the facility had to overtake all organizing tasks in the nursing home, i.e. information of personnel, residents and/or their legal guardians, distribution of information material and collection of the informed consent form, collection and shipment of faecal samples, and ii) at least 10 residents from each facility should be enrolled. Eleven nursing homes from different geographic areas (10 districts around Hesse) and different size (40–120 beds), which were run by different organisations (e.g. German Red Cross, Church, Foundations and privately-run) agreed and were enrolled between June 2010 and May 2011. General information about the facility's structure and history of recent CDI cases was collected by a questionnaire which was filled out by the care manager. Informed consent was obtained from all participants or their legal guardians. Individual conditions of participants were evaluated using a questionnaire that was filled out by the healthcare personnel for each participant.

In parallel, the population outside nursing homes in Hesse was called on to participate in this study as a control group. An information campaign was launched by articles in the local press, posters, flyers, telephone calls, personal visits to institutions including regional public health offices, municipal and state administration, clubs and societies, companies, a hairdressers' saloon, two pharmacies, and a general practitioner's office. Eligible were persons of ≥16 years who were not professionally involved in patient care or worked in diagnostic laboratories. The participants completed a short questionnaire and signed the informed consent form. The study protocol was approved by the Ethic Committee of the General Medical Council of Hesse.

### Collection and transport of stool samples

Faecal samples were collected by the healthcare personnel in the nursing homes. Residents who had agreed to participate, or for whom a written agreement was obtained from the legal guardian, and who were cooperative were enrolled. Samples were stored for maximal one day at 4–8°C and sent to the laboratory of the Hesse State Health Office (HSHO) via courier or with the post. Samples from the general population were either submitted directly to our laboratory or sent with the post (average transport time: one day). The transport conditions did not differ considerably between the groups.

### Laboratory investigation

Clostridium cultures were performed on *C. difficile* selective agar containing cycloserine, cefoxitin and fructose (Oxoid, Wesel, Germany) with and without pre-treatment with ethanol (50% (v/v) final concentration, 1 h, room temperature). The cultures were incubated at 37°C under anaerobic conditions for 5–6 days and examined every 2–3 days. Identification was performed by routine microbiologic techniques and a latex agglutination test for *C. difficile* (Microgen, Cambereley, U.K.) [21]. All isolates were tested for toxin production in vitro by using an ELISA detecting toxin A and B (Biopharm, Darmstadt, Germany) und for the presence of *C. difficile* Toxin B gene (*tcd*B) by using a PCR-hybridisation assay (Hyplex, Gießen, Germany) according to the manufacturer's recommendations.

### PCR-ribotyping

Ribotyping was performed at the Robert Koch Institute in Wernigerode, in the HSHO, or in the Department of Medical Microbiology, Leiden University Medical Center, according the protocol of Bidet et al. [24], except that PCR Products were run on 1.5% agarose gel at 85 volts for 4 hours. Isolates were assigned novel ribotypes (RT) if their patterns differed from previously named patterns by at least one band.

### Statistical analysis

Participants' characteristics were compared with the Chi-square test, Fishers exact test and t-test as appropriate. Point prevalence ratios with accompanying 95% confidence interval (CI) were calculated to assess which characteristics were associated with colonization. Results were considered statistically significant when the 2-sided P value was <0.05. Statistical analysis was performed with PASW Statistics version 17.0 (SPSS Inc., Chicago, USA).

## Supporting Information

Figure S1 **Population pyramid age structure of Hesse and Germany in 2008 as calculated by the Federal Statistical Office (Statistisches Bundesamt), Wiesbaden, Germany.**
(PDF)
