# Supplementary material for: High Prevalence of Clostridium difficile Colonization among Nursing Home Residents in Hesse, Germany
Source: PLoS One. 2012 Jan 11;7(1):e30183. doi: 10.1371/journal.pone.0030183 (PMC3256225; doi:10.1371/journal.pone.0030183)
Supplement: Figure S1 — Population pyramid age structure of Hesse and Germany in 2008 as calculated by the Federal Statistical Office (Statistisches Bundesamt), Wiesbaden, Germany. (PDF) [file pone.0030183.s001.pdf]

12. koordinierte Bevölkerungsvorausberechnung

DESTATIS wissen. nutzen.

Altersaufbau 2008

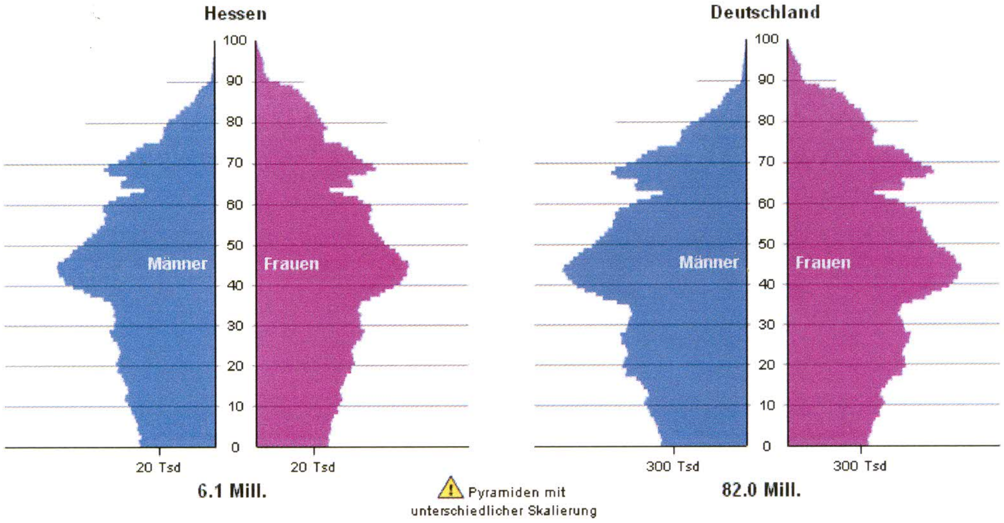

Bundesland	Bevölkerung im Jahr 2008					
	Insgesamt	jünger als 20 Jahre	20 - 64 Jahre	65 - 79 Jahre	80 Jahre und älter	Altenquotient[2]
	Millionen	Prozent[1]				
Baden-Württemberg	10.8	20.4	60.3	14.5	4.7	32
Bayern	12.5	20.0	60.7	14.6	4.8	32
Berlin	3.4	16.4	64.9	14.7	4.0	29
Brandenburg	2.5	15.8	62.3	17.6	4.3	35
Bremen	0.7	17.6	61.1	15.7	5.6	35
Hamburg	1.8	17.3	63.9	13.9	4.8	29
**Hessen**	**6.1**	**19.4**	**60.9**	**14.8**	**4.9**	**32**
Mecklenburg-Vorpommern	1.7	15.6	62.8	17.5	4.2	34
Niedersachsen	7.9	20.5	59.0	15.5	5.0	35
Nordrhein-Westfalen	17.9	20.1	59.8	15.2	4.9	34
Rheinland-Pfalz	4.0	19.8	59.8	15.2	5.1	34
Saarland	1.0	18.0	60.1	16.6	5.3	36
Sachsen	4.2	15.1	60.8	18.4	5.8	40
Sachsen-Anhalt	2.4	14.9	61.4	18.5	5.2	39
Schleswig-Holstein	2.8	20.1	58.8	16.2	5.0	36
Thüringen	2.3	15.1	62.4	17.6	5.0	36
**Deutschland**	**82.0**	**19.1**	**60.6**	**15.5**	**4.9**	**34**

[1] Abweichungen von 100% sind rundungsbedingt.

[2] Die Zahl der 65-Jährigen und Älteren je 100 Personen im Alter von 20 bis unter 65 Jahren.

Anmerkungen

Diese Darstellung basiert auf den Ergebnissen der 12. koordinierten Bevölkerungsvorausberechnung für Deutschland und die Länder, Variante 1-W1 (Untergrenze der "mittleren" Bevölkerung)

Die Annahmen		
	Für Deutschland	Für die Länder
Außenwanderung	Allmählicher Anstieg des Wanderungssaldos bis 2014 auf jährlich +100 000 Personen; ab 2014 konstant	Verteilung des Außenwanderungssaldos nach Ländern entsprechend dem mehrjährigen Durchschnitt
Binnenwanderung	–	Der Anteil der Landesbevölkerung, der in ein anderes Bundesland fortzieht, bleibt bis zum Jahr 2020 konstant. Die Zuzüge eines Bundeslandes ergeben sich als Summe der Fortzüge der übrigen 15 Länder in dieses Land. Zwischen den Jahren 2020 und 2030 nimmt die Binnenwanderung kontinuierlich ab. Ab dem Jahr 2030 bleibt der Saldo der Binnenwanderung bei Null.
Geburtenhäufigkeit	Annähernd konstant bei 1,4 Kindern je Frau	In den alten Ländern annähernde Konstanz bei einem weiteren Anstieg des durchschnittlichen Gebäralters bis 2020; in den neuen Ländern weitere

		Annäherung an die westdeutsche Verteilung der Geburten nach dem Alter der Mutter. Länderspezifische Unterschiede bleiben erhalten.
Lebenserwartung	Basisannahme: Zielwerte für Neugeborene im Jahr 2060:<br>Jungen: 85,0 Jahre<br>Mädchen: 89,2 Jahre	In den alten Ländern gleicher Trend wie für Deutschland insgesamt. In den neuen Ländern zuerst schnellerer Anstieg, dann gleicher Trend wie für Deutschland. Länderspezifische Unterschiede bleiben erhalten.

Weitere Auskünfte gibt

Olga Pötzsch
+49 611 75-3304
olga.poetzsch@destatis.de
Statistisches Bundesamt
65180 Wiesbaden, Deutschland

© Statistisches Bundesamt, Wiesbaden 2010
